# *SF3B1* mutations constitute a novel therapeutic target in breast cancer

**DOI:** 10.1002/path.4483

**Published:** 2014-12-22

**Authors:** Sarah L Maguire, Andri Leonidou, Patty Wai, Caterina Marchiò, Charlotte KY Ng, Anna Sapino, Anne-Vincent Salomon, Jorge S Reis-Filho, Britta Weigelt, Rachael C Natrajan

**Affiliations:** 1 The Breakthrough Breast Cancer Research Centre, The Institute of Cancer ResearchLondon, UK; 2 Department of Molecular Pathology, The Institute of Cancer ResearchLondon, UK; 3 Department of Medical Sciences, University of TurinTurin, Italy; 4 Department of Pathology, Memorial Sloan Kettering Cancer CenterNew York, NY, USA; 5 Institut Curie, Department of Tumour Biology26 Rue d'Ulm, 75248, Paris Cédex 05, France; 6 INSERM U93426 Rue d'Ulm, 75248, Paris Cédex 05, France

**Keywords:** breast cancer, next-generation sequencing, drivers, *SF3B1*, alternative splicing, spliceostatin A

## Abstract

Mutations in genes encoding proteins involved in RNA splicing have been found to occur at relatively high frequencies in several tumour types including myelodysplastic syndromes, chronic lymphocytic leukaemia, uveal melanoma, and pancreatic cancer, and at lower frequencies in breast cancer. To investigate whether dysfunction in RNA splicing is implicated in the pathogenesis of breast cancer, we performed a re-analysis of published exome and whole genome sequencing data. This analysis revealed that mutations in spliceosomal component genes occurred in 5.6% of unselected breast cancers, including hotspot mutations in the *SF3B1* gene, which were found in 1.8% of unselected breast cancers. *SF3B1* mutations were significantly associated with ER-positive disease, *AKT1* mutations, and distinct copy number alterations. Additional profiling of hotspot mutations in a panel of special histological subtypes of breast cancer showed that 16% and 6% of papillary and mucinous carcinomas of the breast harboured the *SF3B1* K700E mutation. RNA sequencing identified differentially spliced events expressed in tumours with *SF3B1* mutations including the protein coding genes *TMEM14C*, *RPL31*, *DYNL11*, *UQCC*, and *ABCC5*, and the long non-coding RNA *CRNDE*. Moreover, *SF3B1* mutant cell lines were found to be sensitive to the SF3b complex inhibitor spliceostatin A and treatment resulted in perturbation of the splicing signature. Albeit rare, *SF3B1* mutations result in alternative splicing events, and may constitute drivers and a novel therapeutic target in a subset of breast cancers. © 2014 The Authors. *The Journal of Pathology* published by John Wiley & Sons Ltd on behalf of Pathological Society of Great Britain and Ireland.

## Introduction

The ability to characterize entire genomes at base pair resolution using massively parallel sequencing technologies provides a unique opportunity to unravel genotypic–phenotypic associations in breast cancer, which can be exploited for the identification of drivers of tumourigenesis and, ultimately, therapeutic targets. Recent breast cancer sequencing studies have highlighted the complex nature of the landscape of breast cancer genomes, characterizing both the mutational signatures of breast cancer [1–3] and their mutational repertoire [4–8]. These seminal studies have highlighted that there are a few highly recurrent mutations in breast cancer, including *TP53* and *PIK3CA*, and a wide spectrum of genes mutated in a small minority of tumours. However, some of the low-frequency mutations (i.e. present in 0.5–2%) of breast cancers constitute bona fide drivers and therapeutic targets in other cancer types, such as *BRAF* and *KRAS* activating hotspot mutations [6].

In addition to known drivers, massively parallel sequencing studies have resulted in the identification of novel mutations in multiple components of the RNA splicing machinery. Somatic mutations affecting different spliceosomal component genes are preferentially found in myeloid neoplasms showing features of myelodysplasia (MDS) and seemingly occur in a mutually exclusive manner [9]. In fact, mutations in the splicing factor 3B subunit 1 gene (*SF3B1*) are rather prevalent in MDS and found to be associated with a distinct phenotype (i.e. the presence of ring sideroblasts) and favourable clinical outcome [10,11]. Mutations in other splicing genes, such as the U2 small nuclear RNA auxiliary factor 1 gene (*U2AF1*), the serine/arginine-rich splicing factor 2 gene (*SRSF2*), and the U2 small nuclear ribonucleoprotein auxiliary factor 35 kDa subunit-related protein 2 gene (*ZRSR2*), have also been reported; however, these are less frequent and not associated with ring sideroblasts in MDS patients [9,10]. More recently, studies have identified mutations in *SF3B1* in subsets of solid tumours from multiple anatomic sites (see ref 11 for a recent review), including 15% of chronic lymphocytic leukaemias (CLLs) [12], 9.7% of uveal melanomas [13], 4% of pancreatic cancers [14], and 1.8% of breast cancers [4–6,8]. Although these mutations have been shown to result in phenotypic changes exemplified by their impact on RNA splicing events in CLLs and uveal melanomas, their impact on outcome seems to vary according to tumour type. Whilst in patients with CLL these mutations are associated with a poor outcome, in patients with uveal melanoma, *SF3B1* mutations are reported to be associated with a good prognosis [12,13,15,16].

Given that mutations affecting spliceosomal component genes have been reported in multiple tumour types, including breast cancer, and may constitute driver events in a subset of cancers, we performed a systematic re-analysis of publicly available exome, whole genome, and RNA sequencing data available for breast cancers. Our aims were to determine whether mutations affecting spliceosomal component genes are associated with specific breast cancer subtypes and, if present, whether these mutations were associated with distinct splicing events and would constitute targets for therapy in these tumours.

## Materials and methods

### Re-analysis of publicly available whole genome and exome massively parallel sequencing datasets

Exome and whole genome sequencing data for 1293 tumours were obtained from The Cancer Genome Atlas (TCGA) and other published studies [3–8]. Processed variant calls reported in these studies were annotated using the Ensembl Variant effect predictor [17] and mutational gene frequencies computed. Binary alignment mapping (BAM) files of *SF3B1* mutant tumours available in TCGA were used to assess the heterozygosity at the *SF3B1* locus using ASCAT [18].

### Tumour samples

Representative frozen or formalin-fixed, paraffin-embedded (FFPE) samples from 65 breast cancers classified as papillary (*n =* 19), mucinous (*n =* 18), and micropapillary (*n =* 28) carcinomas were retrieved from the authors' institutions and surveyed for the presence of the *SF3B1* K700E hotspot mutation (Supplementary Table 1). All cases were reviewed by at least two pathologists (CM, AS, AV-S, and/ or JSR-F) prior to their inclusion in this study. This study was approved by the authors' local research ethics committees. Analyses were performed anonymously.

### Immunohistochemistry

Representative sections of each case were cut at 3 µm and mounted on silane-coated slides. Immunohistochemistry was performed as previously described [19,20], using antibodies raised against oestrogen receptor (ER), progesterone receptor (PR), HER2, and epithelial membrane antigen (EMA). Antibody clones, dilution, antigen retrieval methods, scoring systems, and cut-offs are summarised in Supplementary Table 2. Positive and negative (omission of the primary antibody and IgG-matched serum) controls were included for each immunohistochemical run. The scoring was performed by at least two pathologists (CM, AS, AV-S, and/ or JSR-F).

### Nucleic acid extraction

DNA and/or RNA were extracted after gross dissection of representative frozen or FFPE tissue blocks to ensure that the samples contained more than 60% tumour cells [21,22] (Supplementary methods). RNA quantity and quality were assessed using the Agilent 2100 Bioanalyzer (Agilent Technologies, Santa Clara, CA, USA). Only samples with an RNA integrity number (RIN) greater than 6 were used for RNA-sequencing library construction.

### Copy number analysis

Normalised circular binary segmented (cbs) SNP6 data were retrieved from TCGA for available *SF3B1* mutant and wild-type tumours, matched on a 1 : 2 ratio for ER, PR, HER2 status, and *PIK3CA* and *TP53* mutational status (Supplementary Table 3). SNP6 copy number data were converted into categorical values by applying thresholds as previously described and subjected to a multi-Fisher exact test with adjustment for multiple testing [23].

### Targeted re-sequencing

Targeted DNA sequencing of ten recurrently mutated genes identified in *SF3B1* mutated (*n =* 3) and wild-type (*n =* 16) breast cancers was performed using the Ion Torrent AmpliSeq technology according to the manufacturer's instructions (Life Technologies, Paisley, UK) (Supplementary methods and Supplementary Table 4). Libraries were amplified using 10 ng of DNA per primer pool using the IT AmpliSeq 2.0 kit and sequenced on two 318 chips at a median depth of more than 1000. The Torrent Suite v4.0.2 pipeline was used to align raw reads and identify variants.

### Paired-end massively parallel RNA sequencing

RNA sequencing was performed using 16 ng of ribosomal-depleted RNA of 14 papillary carcinomas of the breast (three *SF3B1* mutant and 11 *SF3B1* wild-type) (Supplementary methods). Four indexed samples were pooled at equimolar concentrations and sequenced on a single lane of a HiSeq 2500 using SBS v3 chemistry (Illumina, San Diego, CA, USA) (2 × 76 cycles). Samples were aligned to the human genome (hg19 build 37) using TopHat version 2.0.8b. Raw counts of reads mapped to genes were calculated using HT-Seq (http://www-huber.embl.de/users/anders/HTSeq/doc/overview.html) and used as input for differential exon usage analysis using DEXSeq [24], with an adjusted *p*-value cut-off of ≤0.1. FASTQ files from available TCGA RNA-sequencing data from *SF3B1* K700E mutant (*n =* 8) and ER, PR, HER2 status, and *PIK3CA* and *TP53* mutational status, and randomly matched controls (*n =* 16) were downloaded from the Cancer Genomics Hub (CGHub; https://cghub.ucsc.edu) and processed as above (TCGA project access number 6223).

Raw targeted re-sequencing and RNA-sequencing data have been deposited in the Sequence Read Archive (SRA) (http://www.ncbi.nlm.nih.gov/sra) and are available under accession numbers PRJNA234087 and PRJNA229096.

### Reverse-transcription PCR (RT-PCR), PCR, and Sanger sequencing validation

Reverse transcription was performed with Superscript III (Invitrogen, Paisley, UK) using 50 ng of RNA per reaction. *SF3B1* K700E mutations were screened by PCR and RT-PCR using KAPA Taq DNA Polymerase (KAPA Biosystems, MA, USA) and sequenced as previously described [22]. Sequences were visualized using 4Peaks (http://4peaks.en.softonic.com/). Primer sequences are listed in Supplementary Table 5.

### Splice variant analysis by quantitative PCR

Primers were designed against predicted alternatively spliced exons using Primer 3 (http://primer3.ut.ee) and retrieved from Furney *et al* [13] (Supplementary Table 5). 1 ng of cDNA was analysed in duplicate to quantify spliced and unspliced forms by real-time quantitative PCR [13]. Briefly, 40 cycles of quantitative reverse-transcription PCR (qRT-PCR) were conducted in 384-well plates using Power SYBR Green reagents (Invitrogen, Paisley, UK) on the ABI 9700HT. The relative abundance of the spliced to unspliced form was calculated using 2^−(CT variant1 mRNA – CT variant2 mRNA)^. For each splicing event, a two-tailed Mann–Whitney *U*-test was applied between *SF3B1* mutated (*n =* 3) and wild-type (*n =* 11) cases.

### Cell line models

Pancreatic Panc 05.04, PANC-1, Capan-1, Capan-2, and endometrial cancer cell lines ESS-1, MFE296, HEC1A, and HEC59 were obtained from ATCC (LGC Standards, UK) and DSMZ (Braunschweig, Germany) and maintained according to recommended growth conditions. The identity of cell lines was confirmed by short tandem repeat (STR) typing using the GenePrint 10 Kit (Promega, UK). *SF3B1* mutant cell lines were identified through COSMIC (http://cancer.sanger.ac.uk/cosmic) and verified by means of Sanger sequencing using the methods and primers described above.

### Short interfering RNA (siRNA)-mediated silencing

*SF3B1* plus eight genes that were consistently differentially spliced in *SF3B1* mutant versus wild-type samples – namely *ABCC5*, *ANKHD1*, *DYNLL1*, *F8*, *RPL31*, *TMEM14C*, *UQCC*, and *CRNDE* – were selected for functional evaluation. Splice variant specific siRNAs were designed to differentially spliced genes that showed large enough unique regions to allow targeting of three or more independent oligos (Supplementary Table 5). Cells were transfected with target and control siRNAs in 96-well plates, using Lipofectamine 2000 (Panc 05.04) or Lipofectamine RNAiMax (Invitrogen). Cell viability was assessed 6–8 days post-transfection as previously described [25], using the CellTiter-Glo® assay (Promega, UK). The cell survival fraction for each siRNA was calculated using the normalised percentage inhibition (NPI) as previously described [26]. Validation of target gene knockdown was performed using qRT-PCR using the delta-delta CT method. Experiments were performed in triplicate.

### Drug sensitivity assays

Cell lines were plated in 96-well plates and 24 h later, media were supplemented with serial dilutions (10^−5^–10^−9^ m) of spliceostatin A (SSA) (Bioquote, York, UK). All experiments were performed in triplicate. Survival was assessed with the CellTiter-Glo® cell viability assay after 7 days of drug treatment. Survival curves and estimated SF_50_ (the drug concentration used following which 50% of cells survive) were calculated using non-linear regression with GraphPad Prism V5.0.

### Statistical analyses

Fisher's exact tests using all collated mutation data were used to determine association of co-occurrence or mutual exclusivity between variables as previously described [27]. The Mann–Whitney *U*-test and Student's *t*-test were employed to compare the mean expression of alternatively spliced exons between *SF3B1* wild-type and mutant tumours, and between drug- and DMSO-treated cells. *p* values less than 0.05 (two-sided) were considered statistically significant.

## Results

### *SF3B1* mutations are associated with ER-positive breast cancer

**Figure 1:**
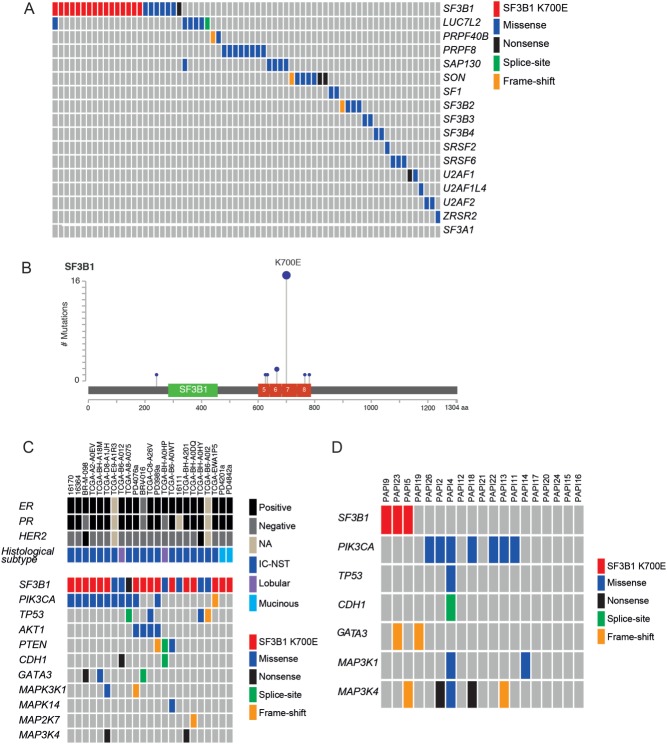
Repertoire of mutations in genes involved in mRNA splicing in breast cancer. (A) Summary of the mutations identified in genes involved in mRNA splicing. Genes are listed on the right-hand side of the diagram and the breast cancer samples across the top. Mutations show a mutually exclusive pattern indicating functional redundancy. Mutation data retrieved from TCGA plus published studies (Nik-Zainal *et al* [3], Banerji *et al* [4], Ellis *et al* [5], TCGA [6], Shah *et al* [7], and Stephens *et al* [8]). (B) Distribution and frequency of *SF3B1* mutations derived from re-analysis of publicly available massively parallel sequencing data from the TCGA breast cancer study and additional studies (as above). Note that mutations are clustered in the HEAT domain (exons 14–16) of the protein with a hotspot point mutation at amino acid 700 (K700E). (C) Histological and molecular status of *SF3B1* mutant samples from the re-analysis. Co-mutation analysis shows *PIK3CA* missense mutations in 47.8% and *AKT1* mutations in 17% of the samples, while mutations in other known driver genes are present at lower frequencies. Note the lack of additional significantly mutated genes or known cancer genes in the two mucinous carcinomas (curated from TCGA, http://www.cbioportal.org/public-portal/study.do?cancer_study_id=brca_tcga_pub, and the Cancer Gene Census, https://cancer.sanger.ac.uk/cosmic). IC-NST = invasive carcinoma of no special type. (D) Repertoire of somatic mutations in 19 papillary carcinomas of the breast, as defined by targeted re-sequencing of known cancer genes. The genes are listed on the left-hand side of the diagram and the breast cancer samples across the top. Two of the *SF3B1* mutant tumours displayed additional mutations affecting *GATA3* and *MAP3K4.*

A systematic re-analysis of exome and whole genome sequencing data from TCGA and other published studies [3–8] identified recurrent mutations in distinct spliceosomal component genes previously reported in other tumour types (Figure 1A, Table 1, and Supplementary Table 6) [9,12,28,29]. Some mutations in spliceosome genes appeared to be mutually exclusive, including *SF3B1* and *PRPF8*, and *SF3B1* and *SON* (odds ratio = 0, *p* < 0.05, Fisher's exact test). Coincident spliceosome mutations were also identified in *SF3B1* and *LUC7L2*, and in *LUC7L2* and *SAP130* in two patients (Figure 1A). Although not significant, mutations in *LUC7L2* and *SAP130* showed a trend towards co-occurrence (odds ratio = 2.883, *p* = 0.9434, Fisher's exact test). Of the most common spliceosome gene mutations (overall frequency > 0.5%), mutations in *SF3B1* were more common in ER-positive breast cancer (2.2%, 21/936 ER-positive versus 0.3%, 1/289 ER-negative, *p* = 0.03914, Fisher's exact test) and mutations in *SAP130* were more common in ER-negative tumours (0.1%, 1/936 ER-positive versus 1.4%, 4/289 ER-negative, *p* = 0.012, Fisher's exact test). Overall, mutations in *SF3B1* were the most common spliceosomal component gene mutation and were identified in 1.8% of unselected breast cancers (23/1293), and occurred as a recurrent hotspot T > C base-pair substitution at codon 700, leading to a glutamic acid from lysine (K700E) in 74% (17/23) of mutant cases (Figure 1B and Table 1). Additional recurrent mutations in *SF3B1* occurred at codon 666 (K666Q and K666E) in 9% of mutant tumours (2/23), and non-recurrent mutations included G241*, N626D, A633V, Y765C, and D781E, which were found in 22% (5/23; Supplementary Table 6). None of mutations were associated with loss of heterozygosity of the wild-type allele (data not shown).

**Table 1:** Summary of spliceosome component mutational frequencies from the re-analysis of public sequencing data (*n =* 1293 tumours*)

Gene symbol	No of cases with mutation	Overall frequency (%)	ER+ (%)	ER−b> (%)	HER2+ (%)	TN (%)
*SF3B1*	23	1.8	2.1	0.3	1.8	0.5
*PRPF8*	8	0.6	0.6	0.7	0.9	0.5
*SON*	9	0.7	0.3	1.4	0.9	1.5
*SAP130*	6	0.5	0.1	1.4	0.9	0.5
*LUC7L2*	7	0.6	0.6	0.3	–	0.5
*SF3B2*	4	0.3	0.2	0.7	1.3	–
*SRSF6*	3	0.2	–	0.7	–	1.0
*ZRSR2*	2	0.2	–	0.7	–	0.5
*U2AF2*	2	0.2	0.1	0.3	0.4	0.5
*SF3B4*	2	0.2	0.1	0.3	0.4	–
*SF3B3*	2	0.2	0.1	0.3	0.4	0.5
*SF1*	2	0.2	0.1	0.3	–	0.5
*PRPF40B*	3	0.2	0.3	–	–	–
*U2AF1L4*	1	0.1	0.1	–	–	–
*U2AF1*	1	0.1	0.1	–	–	–
*SRSF2*	1	0.1	0.1	–	0.4	–

* Samples retrieved from Nik-Zainal *et al* [3], Banerji *et al* [4], Ellis *et al* [5], TCGA [6], Shah *et al* [7], and Stephens *et al* [8].

ER = oestrogen receptor; HER2 + = HER2-positive; TN = triple-negative.

### *SF3B1* mutations are associated with specific genomic alterations

A comparison of the genomic features of *SF3B1* K700E mutant tumours with available phenotypic information (*n =* 16) with those of stage, ER, PR, HER2, *PIK3CA*, and *TP53* mutation status-matched *SF3B1* K700E wild-type tumours (*n =* 32; Supplementary Table 3) revealed that *SF3B1* K700E mutant tumours displayed a higher frequency of chromosomal gains on 16q12–q13 and 16q21–q22 and losses on 1p36–p35, 16q11–q13, and 16q21–q23 (adjusted *p* < 0.05, multi-Fisher's exact test). These observations suggest that *SF3B1* K700E mutant tumours have a distinct repertoire of copy number alterations (Supplementary Figure 1 and Supplementary Table 7). No differences in the frequency of amplifications between *SF3B1* K700E mutant and wild-type tumours were detected. Although no difference was seen in the total number of somatic coding mutations between *SF3B1* K700E mutant and wild-type samples (*p* = 0.597, *t*-test; *SF3B1* K700E mutant average 23.3, range 9–59; *SF3B1* wild-type average 25.59, range 1–85), 56.3% (9/16) of the *SF3B1* mutant samples also carried *PIK3CA* mutations, while mutations in *TP53* were not as prevalent (6.3%, Figure 1C). In addition, *AKT1* hotspot E17K mutations were found to be significantly more frequent in *SF3B1* K700E mutated tumours (4/16) than in matched wild-type cancers (0/32, *p* = 0.009353, Fisher's exact test). Interestingly, 2 out of 24 (8.3%) mucinous carcinomas of the breast annotated in the data were identified as having *SF3B1* K700E mutations and lacked mutations in additional significantly mutated genes or known cancer genes (Figure 1C).

### *SF3B1* K700E mutations are more common in ER-positive special histological types of breast cancer

Given the apparent association of *SF3B1* mutations with mucinous carcinomas of the breast, we screened additional cohorts of ER-positive special histological types of breast cancer for *SF3B1* K700E mutations. Mucinous (*n =* 18), papillary (*n =* 19), and micropapillary (*n =* 28) carcinomas were subjected to Sanger sequencing (Supplementary Table 1). K700E mutations were identified in 6% (1/18), 16% (3/19), and 0% (0/28) of mucinous, papillary, and micropapillary carcinomas, respectively. These mutations were expressed at the RNA level in all papillary carcinomas tested, where matched RNA was available. Although the small number of cases subjected to *SF3B1* mutation profiling could have resulted in a type I or α error resulting in a higher frequency of mutations than the mutation rate in unselected breast cancers, the probability of finding three samples harbouring an *SF3B1* mutation by chance is less than 0.5% (based on a binomial distribution assuming a mutation rate of 1.8% in unselected breast cancers).

Given the higher frequency of *SF3B1* K700E mutations in papillary carcinomas of the breast, we hypothesised that these mutations may underpin their biology and may be present at additional hotspots and/or be sub-clonal in the ‘wild-type’ samples. To address these questions, the 19 papillary carcinomas were subjected to targeted DNA re-sequencing of *SF3B1* exons 14–16 and of nine additional genes, which are recurrently mutated in ER-positive breast cancers harbouring *SF3B1* mutations and have been postulated as drivers [5]. This analysis failed to identify additional or sub-clonal *SF3B1* mutations in the 16 papillary carcinomas that had been shown be *SF3B1* wild-type by Sanger sequencing analysis. Overall, 63% (12/19) of the samples were found to display mutations in additional cancer genes including *PIK3CA* in 37% (7/19), *TP53* in 5% (1/19), *GATA3* in 11% (2/19), *MAP3K1* 11% (2/19), and *MAP3K4* in 21% (4/19) (Figure 1D and Supplementary Table 8). In contrast to *SF3B1* mutant unselected breast cancers, no papillary carcinomas in this series were found to harbour *AKT1* hotspot mutations.

### SF3B1 K700E mutations are associated with differential splicing

**Figure 2:**
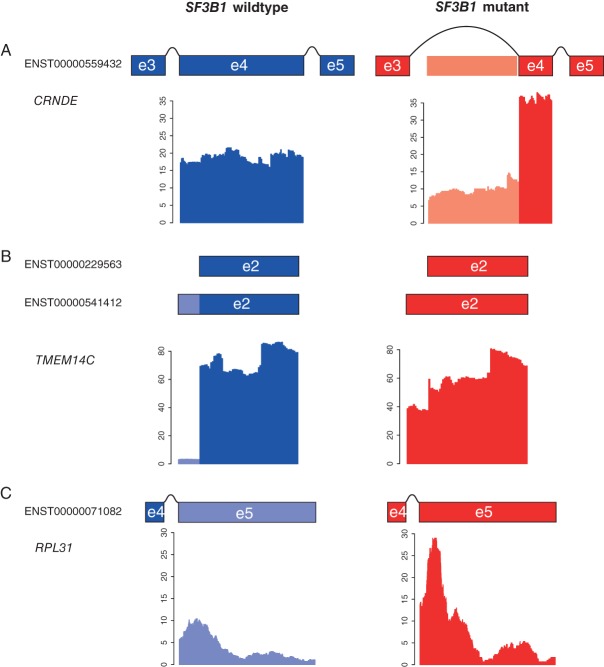
Identification of differential splicing in *SF3B1* mutant (*n =* 3) and wild-type (*n =* 11) papillary carcinomas of the breast. Plots of normalised RNA-sequencing reads for (A) *CRNDE*, (B) *TMEM14C*, and (C) *RPL31* in *SF3B1* wild-type (blue) and *SF3B1* mutant (red) tumours. Schematic representations of the exon structures are shown above the graph, with exons represented by boxes. Differentially spliced exon bins are indicated by lighter coloured shading. Box plots representing the validation of the differential splicing event in wild-type (blue) versus mutant (red) tumours by quantitative RT-PCR.

Given the previous association of *SF3B1* mutations with differential splicing in uveal melanoma [13], we hypothesised that *SF3B1* K700E mutations in breast cancer would also lead to differential splicing. Massively parallel RNA sequencing of *SF3B1* mutant (*n =* 3) versus wild-type (*n =* 11) papillary carcinomas of the breast identified differential exon usage in 122 transcripts (*p* < 0.1 FDR, Supplementary Table 9). In addition, re-analysis of available RNA-sequencing data of (*n =* 8) *SF3B1* K700E mutant and stage, ER, PR, HER2, *PIK3CA*, and *TP53* mutation status-matched *SF3B1* wild-type breast cancers (*n =* 16) from TCGA revealed differential exon usage in 218 transcripts (*p* < 0.1 FDR, Supplementary Table 10). Nine of these genes were found to be in common, and seven showed exact differential exon usage (*TMEM14C*, *RPL31*, *CRNDE*, *DYNLL1, ICA1*, *RPL24*, and *MTERFD3*, *p* = 2.988529e-14, hypergeometric test, Table 2). In addition, four transcripts (ie *TMEM14C*, *RPL31*, *CRNDE*, and *DYNLL1*) were found in common with uveal melanoma [13] (*p* = 7.047575e-11, hypergeometric test) and these showed identical differential splicing in papillary carcinomas of the breast, invasive breast carcinomas of no special type, and uveal melanomas displaying *SF3B1* mutations (Figure 2 and Table 2).

**Table 2:** Alternative gene splicing associated with *SF3B1* mutations in breast cancer

Gene symbol	Gene ID	This study (DEXSeq)	TCGA (DEXSeq)	Furney *et al* [13]	Validated qRT-PCR
*TMEM14C*	ENSG00000111843	✓	✓	✓	✓
*RPL31*	ENSG00000071082	✓	✓	✓	✓
*CRNDE*	ENSG00000245694	✓	✓	✓	✓
*DYNLL1*	ENSG00000088986	✓	✓	✓	–
*MZB1*	ENSG00000170476	✓	✓	–	–
*ICA1*	ENSG00000003147	✓	✓	–	–
*RPL24*	ENSG00000114391	✓	✓	–	–
*MTERFD3*	ENSG00000120832	✓	✓	–	–
*OBSL1*	ENSG00000124006	✓	✓	–	–
*ABCC5*	ENSG00000114770	–	–	✓	✓
*UQCC*	ENSG00000101019	–	–	✓	✓
*GUSBP11*	ENSG00000228315	–	–	✓	✓
*ANKHD1*	ENSG00000131503	–	–	✓	✓
*ADAM12*	ENSG00000148848	–	–	✓	✓
*F8*	ENSG00000185010	–	–	✓	NS
*GAS8*	ENSG00000141013	–	–	✓	NS

NS = not significant (not validated).

We next additionally tested alternative splicing of seven genes shown to be differentially spliced from Furney *et al* [13] (*GUSBP11*, *UQCC*, *ANKHD1*, *GAS8*, *F8*, *ADAM12*, and *ABCC5*) using qRT-PCR in *SF3B1* mutant and wild-type papillary carcinomas. This analysis identified *UQCC*, *F8*, *ABCC5*, and *GUSBP11* to be differentially spliced in papillary carcinomas, whereas *GAS8* and *ADAM12* showed no difference (Supplementary Figure 2). Our results suggest that *SF3B1* mutations are associated with alternative splicing of key genes in ER-positive breast cancer that are independent of tumour type.

### *SF3B1* mutant cells are sensitive to SF3b inhibition

**Figure 3:**
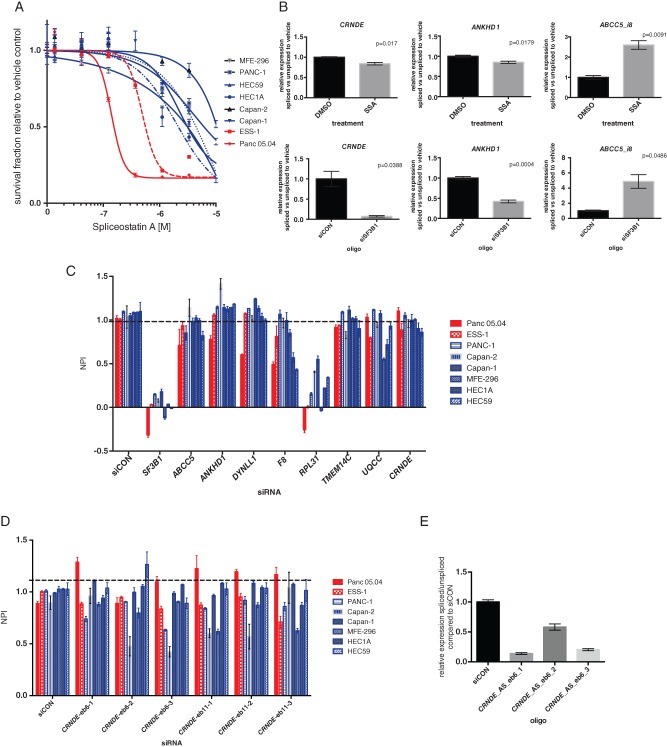
*SF3B1* mutant cells are sensitive to SF3b inhibition. (A) Dose–response curves of *SF3B1* mutant cancer cell lines (red) or wild-type (blue) to the SF3b complex inhibitor spliceostatin A (*p* = 0.0118, *t*-test). (B) Bar plots of the relative expression of alternative spliced transcripts in ESS-1 cells upon 12 h spliceostatin A (SSA) treatment (SF30 concentration) or SF3B1 siRNA-mediated silencing relative to vehicle (DMSO) or non-targeting controls (siCON). Error bars represent the standard error of the mean of three replicates. (C) Bar plots illustrating the normalised percentage inhibition (NPI) relative to siCON negative and ubiquitin B positive controls of cells following transfection with SMARTpool siRNA of *ABCC5*, *ANKHD1*, *DYNLL1*, *F8*, *RPL31*, *TMEM14C*, *UQCC*, and *CRNDE* in *SF3B1* mutant (red) or wild-type (blue) cancer cells. Error bars represent the standard error of the mean of three replicates. None of the genes showed a significant difference in cell viability between mutant and wild-type cells (*p* > 0.05, *t*-test). (D) Bar plots illustrating the normalised percentage inhibition (NPI) relative to siCON negative and ubiquitin B positive controls of cells following transfection with custom siRNA oligos against the *CRNDE* alternative spliced isoform (eb6) in *SF3B1* mutant (red) or wild-type (blue) cancer cells. Error bars represent the standard error of the mean of three replicates. None of the genes showed a significant difference in cell viability between mutant and wild-type cells (*p* > 0.05, *t*-test). (E) Bar plot showing qPCR validation of siRNA oligos against the *CRNDE* alterative spliced isoform (eb6) relative to siCON.

Based on our observations that *SF3B1* mutant tumours display a conserved splicing signature, we posited that differential splicing of the downstream targets may drive tumour growth. Treatment of two *SF3B1* mutant cell lines, Panc 05.04 (pancreatic, K700E) and ESS-1 (endometrial, K666N), and six wild-type controls (three pancreatic and three endometrial) with an SF3b complex inhibitor, spliceostatin A, revealed that *SF3B1* mutant cell lines were significantly more sensitive to this agent (*p* = 0.0118, *t*-test, Figure 3A). Moreover, treatment with spliceostatin A resulted in significant perturbation of the splicing signature, in a similar manner to SF3B1 siRNA-mediated knockdown (Figure 3C). In addition, siRNA-mediated silencing of all transcripts of eight genes that showed a consistent differential splicing signature across multiple cancer types (*ABCC5*, *ANKHD1*, *DYNLL1*, *F8*, *RPL31*, *TMEM14C*, *UQCC*, and *CRNDE*) failed to reveal differences in cell viability (Figure 3C). In addition, silencing of specific splice variants of *CRNDE* failed to identify any *SF3B1* mutant-specific effects (Figures 3D and 3E). These results suggest that *SF3B1* mutations are likely driving the malignant phenotype of the cells through perturbations in RNA splicing; however, this driving effect could not be ascribed to a single differentially spliced transcript tested.

Taken together, our findings demonstrate that although *SF3B1* mutations are unlikely to account for the histological characteristics of papillary carcinomas of the breast, these mutations affect mRNA splicing and likely constitute driver genetic events.

## Discussion

Here we describe the repertoire of mutations in spliceosomal components in breast cancer through a re-analysis of public exome and whole genome sequencing data. Our data reveal that some spliceosome mutations are mutually exclusive in breast cancer, consistent with the observations made in myelodysplastic lesions [9]; however, a small proportion of tumours harboured mutations in more than one spliceosome component. Moreover, we demonstrate that *SF3B1* is the most commonly mutated spliceosomal component gene in breast cancer and that mutations affecting this gene are significantly associated with ER-positive disease. In addition, *SF3B1* mutations are found to be associated with specific genomic alterations including concurrent *AKT1* hotspot mutations and a lower frequency of the hallmark chromosomal aberrations reported in ER-positive IC-NST (ie loss of 16q) akin to mucinous carcinomas of the breast [23]. Our exploratory, hypothesis-generating analysis of special histological types of breast cancer provides evidence to suggest that the codon 700 *SF3B1* hotspot mutations may be more prevalent in mucinous and papillary carcinomas of the breast than in usual types of breast cancer of the same grade, ER, and HER2 status. Targeted re-sequencing of our cohort of 19 papillary carcinomas of the breast failed to identify any sub-clonal mutations in additional tumours, indicating that these mutations, when present, are found in the modal clone of the lesions, but are unlikely to account for the papillary histological pattern.

The *SF3B1* gene encodes subunit 1 of splicing factor 3b, a component of the U2 small nuclear ribonucleoprotein, which is involved in catalysing precursor mRNA to mature transcripts. SF3B1 contains several HEAT domains (Huntingtin, Elongation factor 3, protein phosphatase 2A, Target of rapamycin 1), which are the hotspots for the majority of the somatic mutations previously documented [10,13,15]. Studies have highlighted that primary tumours with *SF3B1* mutations display alternative splicing in selected key genes in CLL [12], MDS [30], and uveal melanoma [13], and that this signature is conserved between cancer sites and is independent of the mutant amino acid [16]. Using a combination of RNA sequencing and qRT-PCR, we identified eight differentially spliced transcripts between mutant *SF3B1* and wild-type breast cancers that were also reported to be differentially spliced in *SF3B1* mutant melanomas [13], highlighting that this signature is additionally conserved in breast cancer.

Given that the spliceosome SF3b complex has emerged as a potential therapeutic target [31], we sought to define whether cancer models harbouring *SF3B1* hotspot mutations would be sensitive to chemical inhibition of SF3B1and RNA-interference silencing of SF3B1 and genes abnormally spliced in *SF3B1* mutant tumours [32]. Here we demonstrate that treatment of *SF3B1* mutant and wild-type cell lines with spliceostatin A resulted in selective inhibition of the growth of mutant cells and in the disruption of the conserved splicing signature. Interestingly, single gene RNAi-mediated silencing of key differentially spliced genes had no effect on cell viability. Although it is plausible that the differentially spliced genes investigated in this study did not account for the dependency of *SF3B1* mutant cells on aberrant splicing, it is conceivable that the differences in pre-mRNA splicing act in concert to produce a cell survival advantage. Given the difficulties in silencing multiple genes systematically, this remains to be tested.

In conclusion, our findings demonstrate that spliceosomal mutations occur in a mutually exclusive manner in breast cancer and that distinct components of the spliceosome are targeted by somatic mutations in different types of breast cancer (eg *SF3B1* and *SAP130* were found to be preferentially mutated in ER-positive and ER-negative disease, respectively). We have demonstrated that *SF3B1* K700E mutations are more frequently found in some special histological types of breast cancer and have provided direct evidence that these are associated with consistent differential splicing patterns in breast cancer. Finally, our study emphasizes the importance of driver genetic alterations found in minor subgroups of breast cancer, given that *SF3B1* mutant cells were shown to be selectively sensitive to a potent SF3b complex inhibitor, spliceostatin A, suggesting that inhibition of the spliceosome complex may constitute a novel therapeutic strategy for patients with *SF3B1* mutations independent of tumour type.

## SUPPORTING INFORMATION ON THE INTERNET

The following supporting information may be found in the online version of this article:

Supplementary methods.

**Figure S1.** Copy number alterations in *SF3B1* K700E mutant (*n =* 8) and wild-type tumours (*n =* 16).

**Figure S2.** Quantitative RT-PCR validation of alternative splicing in *SF3B1* K700E mutant (*n =* 3) versus wild-type (*n =* 11) papillary carcinomas of the breast.

**Table S1.** Details of independent breast cancer patient cohorts.

**Table S2.** Summary of antibodies, clones, dilutions, antigen retrieval methods, scoring, and cut-offs used.

**Table S3.** Phenotype characteristics of matched tumours from TCGA and other public data.

**Table S4.** Summary of coverage for Custom Ampliseq library.

**Table S5.** Primer and custom siRNA oligonucleotide sequences.

**Table S6.** List of mutations in spliceosomal component genes from re-analysis of public exome and whole genome sequencing data.

**Table S7.** Chromosomal regions of recurrent copy number changes significantly associated with *SF3B1* mutant breast cancers, and the protein coding genes, microRNAs, and copy number variations mapping to these regions.

**Table S8.** List of variants identified from targeted re-sequencing of papillary carcinomas of the breast (*n =* 19).

**Table S9.** List of significantly differentially spliced genes from the DEXSeq analysis of *SF3B1* mutant (*n =* 3) and wild-type papillary breast carcinomas (*n =* 11).

**Table S10.** List of significantly differentially spliced genes from the DEXSeq analysis of *SF3B1* mutant (*n =* 8) and invasive breast carcinomas of no special type from TCGA (*n =* 16).

## References

[1] Alexandrov LB, Nik-Zainal S, Wedge DC (2013). Signatures of mutational processes in human cancer. Nature.

[2] Burns MB, Lackey L, Carpenter MA (2013). APOBEC3B is an enzymatic source of mutation in breast cancer. Nature.

[3] Nik-Zainal S, Alexandrov LB, Wedge DC (2012). Mutational processes molding the genomes of 21 breast cancers. Cell.

[4] Banerji S, Cibulskis K, Rangel-Escareno C (2012). Sequence analysis of mutations and translocations across breast cancer subtypes. Nature.

[5] Ellis MJ, Ding L, Shen D (2012). Whole-genome analysis informs breast cancer response to aromatase inhibition. Nature.

[6] The Cancer Genome Atlas Network (2012). Comprehensive molecular portraits of human breast tumours. Nature.

[7] Shah SP, Roth A, Goya R (2012). The clonal and mutational evolution spectrum of primary triple-negative breast cancers. Nature.

[8] Stephens PJ, Tarpey PS, Davies H (2012). The landscape of cancer genes and mutational processes in breast cancer. Nature.

[9] Yoshida K, Sanada M, Shiraishi Y (2011). Frequent pathway mutations of splicing machinery in myelodysplasia. Nature.

[10] Papaemmanuil E, Cazzola M, Boultwood J (2011). Somatic *SF3B1* mutation in myelodysplasia with ring sideroblasts. N Engl J Med.

[11] Scott LM, Rebel VI (2013). Acquired mutations that affect pre-mRNA splicing in hematologic malignancies and solid tumors. J Natl Cancer Inst.

[12] Quesada V, Conde L, Villamor N (2012). Exome sequencing identifies recurrent mutations of the splicing factor *SF3B1* gene in chronic lymphocytic leukemia. Nature Genet.

[13] Furney SJ, Pedersen M, Gentien D (2013). *SF3B1* mutations are associated with alternative splicing in uveal melanoma. Cancer Discov.

[14] Biankin AV, Waddell N, Kassahn KS (2012). Pancreatic cancer genomes reveal aberrations in axon guidance pathway genes. Nature.

[15] Harbour JW, Roberson ED, Anbunathan H (2013). Recurrent mutations at codon 625 of the splicing factor *SF3B1* in uveal melanoma. Nature Genet.

[16] Gentien D, Kosmider O, Nguyen-Khac F (2014). A common alternative splicing signature is associated with *SF3B1* mutations in malignancies from different cell lineages. Leukemia.

[17] McLaren W, Pritchard B, Rios D (2010). Deriving the consequences of genomic variants with the Ensembl API and SNP Effect Predictor. Bioinformatics.

[18] Van Loo P, Nordgard SH, Lingjaerde OC (2010). Allele-specific copy number analysis of tumors. Proc Natl Acad Sci U S A.

[19] Marchio C, Iravani M, Natrajan R (2009). Mixed micropapillary–ductal carcinomas of the breast: a genomic and immunohistochemical analysis of morphologically distinct components. J Pathol.

[20] Marchio C, Iravani M, Natrajan R (2008). Genomic and immunophenotypical characterization of pure micropapillary carcinomas of the breast. J Pathol.

[21] Manie E, Vincent-Salomon A, Lehmann-Che J (2009). High frequency of *TP53* mutation in *BRCA1* and sporadic basal-like carcinomas but not in *BRCA1* luminal breast tumors. Cancer Res.

[22] Natrajan R, Wilkerson PM, Marchio C (2014). Characterization of the genomic features and expressed fusion genes in micropapillary carcinomas of the breast. J Pathol.

[23] Lacroix-Triki M, Suarez PH, MacKay A (2010). Mucinous carcinoma of the breast is genomically distinct from invasive ductal carcinomas of no special type. J Pathol.

[24] Anders S, Reyes A, Huber W (2012). Detecting differential usage of exons from RNA-seq data. Genome Res.

[25] Natrajan R, Mackay A, Wilkerson PM (2012). Functional characterization of the 19q12 amplicon in grade III breast cancers. Breast Cancer Res.

[26] Brough R, Frankum JR, Sims D (2011). Functional viability profiles of breast cancer. Cancer Discov.

[27] Gao J, Aksoy BA, Dogrusoz U (2013). Integrative analysis of complex cancer genomics and clinical profiles using the cBioPortal. Sci Signal.

[28] Rossi D, Bruscaggin A, Spina V (2011). Mutations of the *SF3B1* splicing factor in chronic lymphocytic leukemia: association with progression and fludarabine-refractoriness. Blood.

[29] Makishima H, Visconte V, Sakaguchi H (2012). Mutations in the spliceosome machinery, a novel and ubiquitous pathway in leukemogenesis. Blood.

[30] Wang L, Lawrence MS, Wan Y (2011). *SF3B1* and other novel cancer genes in chronic lymphocytic leukemia. N Engl J Med.

[31] Kaida D, Motoyoshi H, Tashiro E (2007). Spliceostatin A targets SF3b and inhibits both splicing and nuclear retention of pre-mRNA. Nature Chem Biol.

[32] Bonnal S, Vigevani L, Valcarcel J (2012). The spliceosome as a target of novel antitumour drugs. Nature Rev Drug Discov.

