# Supplementary material for: SF3B1 mutations constitute a novel therapeutic target in breast cancer
Source: J Pathol. 2014 Dec 22;235(4):571–80. doi: 10.1002/path.4483 (PMC4643177; doi:10.1002/path.4483)
Supplement: Supplementary file 1 — Supplementary methods. [file path0235-0571-sd1.doc]

**SUPPORTING INFORMATION**

**Supplementary methods**

**Nucleic acid extraction**

From frozen samples, DNA and/or RNA were extracted after gross dissection of representative frozen tissue blocks to ensure that the samples contained >60% of tumor cells as previously described [20,21]. From FFPE samples, representative tumor blocks were micro-dissected with a sterile needle under a stereomicroscope (Olympus SZ61, Tokyo, Japan) to ensure a percentage of tumor cells greater than 80% as previously described [20]. DNA and RNA were extracted using standard protocols [21]. DNA samples were quantified using Qubit fluorometric quantification (Invitrogen, Paisley, UK). RNA quantity and quality were assessed using the Agilent 2100 Bioanalyzer. Only samples with a RNA integrity number (RIN) >6 were used for RNA-sequencing library construction.

**Targeted re-sequencing**

Targeted DNA sequencing of ten recurrently mutated genes identified in *SF3B1* mutated breast cancers was performed using the Ion Torrent AmpliSeq technology (Life Technologies, Paisley UK) in 19 papillary carcinomas of the breast. Custom AmpliSeq libraries were designed using the Ion AmpliSeq Designer (https://www.ampliseq.com/, Life Technologies), resulting in 287 amplicons distributed between in 2 primer pools (Supplementary Table S9). Libraries were amplified using 10ng of DNA per primer pool using the IT AmpliSeq 2.0 kit (Life Technologies) following the manufacturer’s instructions. Barcoded libraries were quantified using Ion Library Quantitation Kit (Life Technologies), diluted to 100pM and pooled together. 4μl of the pool was used for processing with the Ion PGM Template OT2 Kit (Life Technologies). Samples were sequenced on two 318 chips, 10 samples per chip with Ion PGM Sequencing 200 Kit v2. 300,000-750,000 reads were obtained per sample with median depth >1,000 for all samples. The Torrent Suite v4.0.2 pipeline (Life Technologies) was used to align raw reads and identify variants.

**Paired-end massively parallel RNA sequencing**

RNA-sequencing was performed using ribosomal-depleted RNA of 14 papillary carcinomas of the breast (3 SF3B1 mutant and 11 SF3B1 wild-type). 100ng of total RNA was depleted of ribosomal RNA using Ribo-Zero (Epicentre, Cambridge, UK). cDNA libraries were then constructed using the NEB Next Ultra directional RNA library prep kit (New England Biolabs, MA, USA) from 16ng of rRNA depleted RNA, according to the manufacturer's instructions. The indexed RNA-seq libraries were quantified using KAPA library quantification (KAPA biosystems, MA, USA) and 4 samples were then pooled together in equal concentrations. The pools were hybridized with one pool per lane, at a concentration of 15pM, to a v3 paired-end flow cell and clonally amplified by bridge amplification on the Illumina cBot. The flow cell was loaded on a HiSeq 2500 and sequenced using SBS v3 chemistry (Illumina) for a total of 161 cycles (two reads of 76 cycles and one indexing read of 9 cycles). Samples were aligned to the human genome (hg19 build 37) using TopHat version 2.0.8b. Reads mapping to two or more locations were removed from analysis. This resulted in an average of 44.5 million reads per sample.. Raw counts of reads mapped to genes were calculated using HT-Seq (http://www-huber.embl.de). These were used as input for differential exon usage analysis using DEXSeq, with an adjusted P-value cut-off of ≤0.10 [23]. FASTQ files from available TCGA RNA-sequencing data from *SF3B1* K700E mutant (n=8) and ER, PR, HER2 status, *PIK3CA* and *TP53* mutational status, and randomly matched controls (n=16) were downloaded from the Cancer Genomics Hub (CGHub, https://cghub.ucsc.edu) and processed as described above (TCGA project access number 6223).

**Short interfering RNA (siRNA)-mediated silencing**

*SF3B1* plus eight genes that were consistently differentially spliced in *SF3B1* mutant versus wild-type samples were selected for functional evaluation, namely *ABCC5*, *ANKDH1*, *DYNL11*, *F8*, *RPL31*, *TMEM14C*, *UQCC* and *CRNDE*. Each gene was targeted with four distinct siRNAs (siGENOME SMARTpool) obtained from Thermo Scientific (Epsom, UK). siGENOME Non-Targeting siRNA Pool #1 and #2 (siCON, D-001206-13 and D-001206-14) were used as non-targeting controls. Splice variant specific siRNA’s were designed to differentially spliced genes that showed large enough unique regions to allow targeting of 3 or more independent oligos. Oligos were designed using the siDESIGN Center (http://dharmacon.gelifesciences.com/design-center/) to cover only sequence specific retained in either *SF3B1* mutant or wild-type tumors (Supplementary Table S5). As a positive control (i.e. a gene whose silencing is lethal) we employed siRNA pools for Ubiquitin B as previously described [24]. Cells were transfected with target and control siRNAs (50nmol/L per well in 100μL total volume) in 96 well plates, using Lipofectamine 2000 (PANC05.04) or Lipofectamine RNAiMax (Invitrogen, Paisley UK) according to manufacturers’ instructions as previously described [24]. 1000-3000 cells were seeded per well that yielded 80-90% confluency in the controls at 6-8 days [24]. Media were replaced every 2 days and cell viability was assessed using the CellTiter-Glo® assay (Promega, Southampton, UK). The cell survival fraction for each siRNA was calculated using the normalized percentage inhibition (NPI) to account for differences in transfection efficiency as described [25]. Validation of target gene knockdown was performed using quantitative RT-PCR relative to β-actin using Sybr Green or Taqman assays (Life Technologies) (DYNLL1 and SF3B1) using the delta CT method as described [24]. Experiments were performed in triplicate.

**References**

**(Note: reference numbers correspond to reference list in main article)**

20. Marchio C, Iravani M, Natrajan R, *et al.* Genomic and immunophenotypical characterization of pure micropapillary carcinomas of the breast. *J Pathol* 2008; **215:** 398–410.

21. Manie E, Vincent-Salomon A, Lehmann-Che J, *et al.* High frequency of *TP53* mutation in *BRCA1* and sporadic basal-like carcinomas but not in *BRCA1* luminal breast tumors. *Cancer Res* 2009; **69:** 663–671.

23. Lacroix-Triki M, Suarez PH, MacKay A, *et al.* Mucinous carcinoma of the breast is genomically distinct from invasive ductal carcinomas of no special type. *J Pathol* 2010; **222:** 282–298.

24. Anders S, Reyes A, Huber W. Detecting differential usage of exons from RNA-seq data. *Genome Res* 2012; **22:** 2008–2017.

25. Natrajan R, Mackay A, Wilkerson PM, *et al.* Functional characterization of the 19q12 amplicon in grade III breast cancers. *Breast Cancer Res* 2012; **14:** R53.
