# Supplementary figures and images for: SF3B1 mutations constitute a novel therapeutic target in breast cancer
Source: J Pathol. 2014 Dec 22;235(4):571–80. doi: 10.1002/path.4483 (PMC4643177; doi:10.1002/path.4483)

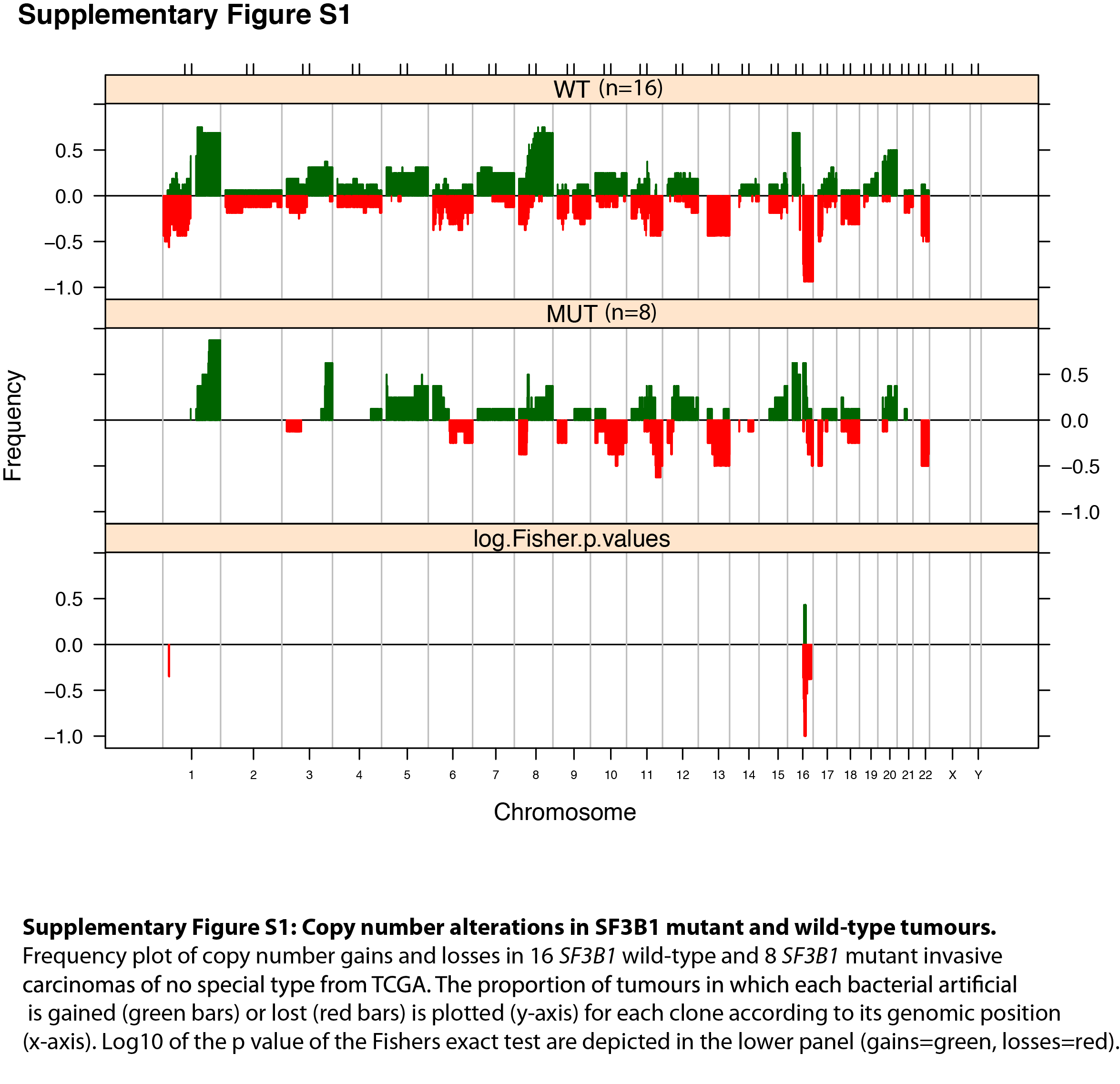

Supplement: Supplementary file 2 — Figure S1. Copy number alterations in SF3B1 K700E mutant (n = 8) and wild-type tumours (n = 16). [file path0235-0571-sd2.tif]

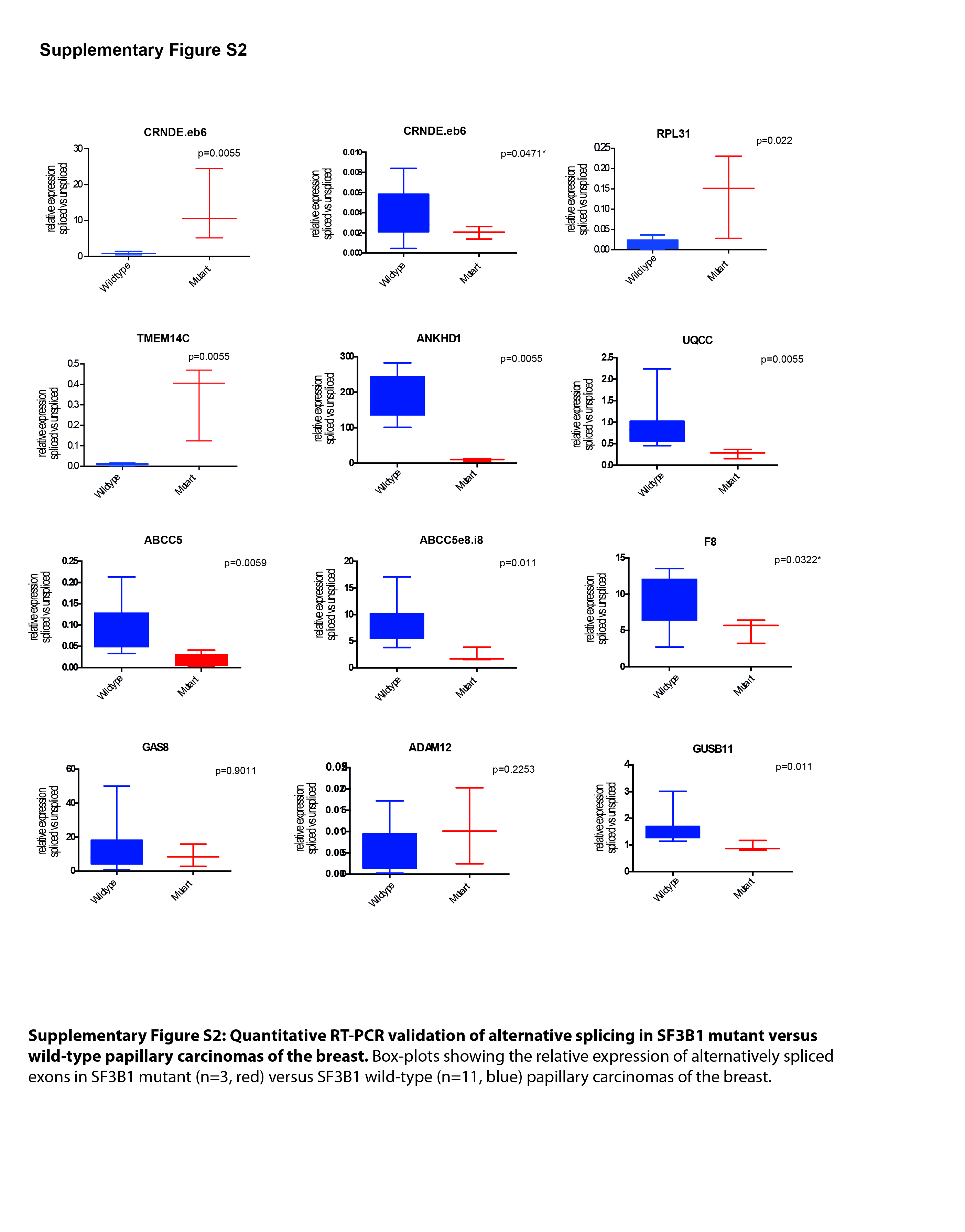

Supplement: Supplementary file 3 — Figure S2. Quantitative RT-PCR validation of alternative splicing in SF3B1 K700E mutant (n = 3) versus wild-type (n = 11) papillary carcinomas of the breast. [file path0235-0571-sd3.tif]
